# Effect of Noisy Galvanic Vestibular Stimulation on Ocular Vestibular-Evoked Myogenic Potentials to Bone-Conducted Vibration

**DOI:** 10.3389/fneur.2017.00026

**Published:** 2017-02-03

**Authors:** Shinichi Iwasaki, Shotaro Karino, Teru Kamogashira, Fumiharu Togo, Chisato Fujimoto, Yoshiharu Yamamoto, Tatsuya Yamasoba

**Affiliations:** ^1^Faculty of Medicine, Department of Otolaryngology, The University of Tokyo, Bunkyo-ku, Tokyo, Japan; ^2^Educational Physiological Laboratory, Graduate School of Education, The University of Tokyo, Bunkyo-ku, Tokyo, Japan

**Keywords:** galvanic vestibular stimulation, stochastic resonance, vestibular-evoked myogenic potentials, vestibulo-ocular reflex, otolith organ

## Abstract

**Objective:**

Galvanic vestibular stimulation (GVS) delivered as zero-mean current noise (noisy GVS) has been shown to improve static and dynamic postural stability probably by enhancing vestibular information. The purpose of this study was to examine the effect of an imperceptible level noisy GVS on ocular vestibular-evoked myogenic potentials (oVEMPs) in response to bone-conducted vibration (BCV).

**Materials and methods:**

oVEMPs to BCV were measured during the application of white noise GVS with an amplitude ranging from 0 to 300 µA [in root mean square (RMS)] in 20 healthy subjects. Artifacts in the oVEMPs caused by GVS were reduced by inverting the waveforms of noisy GVS in the later half of the stimulus from the one in the early half. We examined the amplitudes of N1 and N1–P1 and their latencies.

**Results:**

Noisy GVS significantly increased the N1 and N1–P1 amplitudes (*p* < 0.05) whereas it had no significant effects on N1 or P1 latencies (*p* > 0.05). Noisy GVS had facilitatory effects in 79% of ears. The amplitude of the optimal stimulus was 127 ± 14 µA, and it increased the N1 and N1–P1 amplitude by 75.9 ± 15% and 47.7 ± 9.1%, respectively, as compared with 0 µA session (*p* < 0.05).

**Conclusion:**

Noisy GVS can increase the amplitude of oVEMPs to BCV in healthy subjects probably *via* stochastic resonance. The results of the present study suggest that noisy GVS may improve static and dynamic postural stability by enhancing the function of the vestibular afferents.

## Introduction

The vestibular labyrinth, which is composed of three semicircular canals and two otolith organs in each ear, senses angular and linear movement of the head, thereby contributing to stabilization of body balance (1). Bilateral dysfunction of the vestibular labyrinth results in dizziness, oscillopsia, and postural instability while moving the head or body.

Galvanic vestibular stimulation (GVS) modulates activity in vestibular hair cells and their afferents by delivering electrical current subcutaneously through electrodes placed over the mastoid bones (2, 3). GVS has been used to probe the vestibular system and its effects on posture and gait (4). It has been shown that GVS delivered as zero-mean current noise (noisy GVS) of an imperceptible magnitude improves various functions, such as the baroreflex function in healthy subjects (5), and autonomic and motor functions in patients with multisystem atrophy and Parkinson’s disease (6, 7). Recently, we have shown that noisy GVS can improve postural stability in healthy subjects as well as in patients with bilateral vestibular dysfunction (8).

The rationale behind these ameliorating effects of noisy GVS is considered to be stochastic resonance (SR), which is a phenomenon wherein the response of a non-linear system to a weak periodic input signal is optimized by the presence of a particular level of noise (9, 10). In this phenomenon, an optimal amount of added noise results in the maximum enhancement, whereas further increase in the noise intensity only degrades detectability or information content. Previous studies have shown that an appropriate intensity of noise can magnify detection of weak subthreshold signals in a variety of sensory information such as visual (11) and auditory perception (12, 13). However, it has not been tested whether noisy GVS can really intensify vestibular information.

In the present study, we investigated the effects of noisy GVS on ocular vestibular-evoked myogenic potentials (oVEMPs) to bone-conducted vibration (BCV). oVEMPs reflect the function of otolith-ocular reflex, especially originated from the utricle (14, 15). The purpose of this study was to see whether SR-like phenomenon can be observed in the vestibulo-ocular reflex.

## Materials and Methods

### Subjects

Twenty healthy subjects (10 men, 10 women; age 25–60 years, mean age 42 ± 2.6 years) were recruited. None of the subjects reported any auditory, vestibular, neurologic, cardiovascular, or orthopedic disorders. All subjects gave written informed consent. All procedures were in accordance with the Declaration of Helsinki and were approved by the University of Tokyo Human Ethics Committee (no. 3379) and registered in UMIN-CTR (UMIN00000829).

### oVEMPs to BCV

The methods for recording oVEMPs to BCV have been described in detail elsewhere (14, 15). In brief, with the subject in a supine position, EMG electrodes were placed on the skin 1 cm below (active) and 3 cm below (indifferent) the center of each lower eyelid. The ground electrode was placed on the chin. During testing, the subject looked up approximately 30° above straight ahead and maintained their focus on a small dot approximately 1 m from their eyes. The signals were amplified by a differential amplifier (bandwidth: 0.5–500 Hz), and the unrectified signals were averaged (*n* = 50) using Neuropack Σ (Nihon Kohden, Tokyo, Japan).

**Figure 1:**
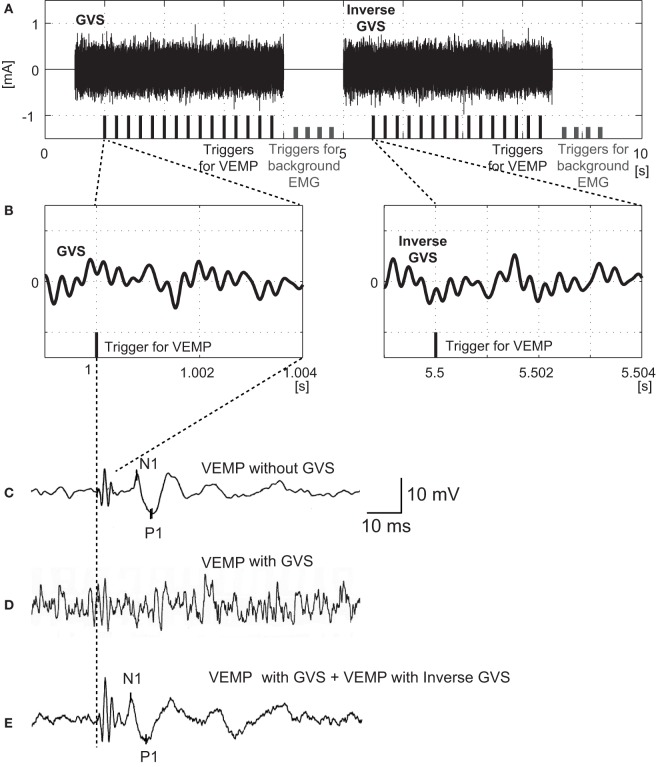
**Protocols for application of noisy galvanic vestibular stimulation (GVS) and recording ocular vestibular-evoked myogenic potentials (oVEMPs) in response to bone-conducted vibration (BCV)**. **(A)** Schema of GVS and triggers for oVEMP measurement. During the early part of GVS (from 0.5 to 4 s) band noise between 1 and 4000 Hz with a flat spectrum was employed. In this example, the amplitude was 200 µA root mean square (RMS). During the later part of GVS (from 5 to 8.5 s), the waveform was inversed. The black bars at the bottom indicate the 15 triggers in each part for averaging oVEMPs. The short gray bars indicate the eight triggers used for averaging background EMG in the absence of BCV or GVS. **(B)** Magnified waveforms of GVS in the neighborhood of the oVEMP trigger. Note that the GVS in the later part is inversed. The 4 ms period after the trigger corresponds to the BCV stimulus duration for evoking oVEMPs. **(C)** oVEMP responses without GVS. **(D)** oVEMP responses after 15 triggers in the former part of noisy GVS (200 µA RMS). **(E)** Averaged oVEMP responses after 30 triggers (15 triggers in the early part and 15 triggers in the later part) of noisy GVS (200 µA RMS).

The BCV stimuli were 4 ms tone-bursts of 500 Hz vibration delivered by a handheld 4810 mini-shaker (Bruel and Kjaer, Naerum, Denmark) fitted with a short rod terminated in a bakelite cap 1.5 cm in diameter, which was placed, without pressure, perpendicularly on the forehead at the hairline in the midline (Fz). The driving voltage was 8.0 V peak to peak, and it produced a peak force level of 128 dB re 1 μN. This BCV caused a linear acceleration in the interaural axis at the mastoids with a maximal acceleration of approximately 0.4 g peak to peak as measured by linear accelerometers placed on the skin over the mastoid. The stimuli were applied five times per second, and the time window for analysis was 50 ms. Responses to 30 stimuli, which was composed of 15 stimuli in the early part and 15 stimuli in the later part, were averaged (Figure 1).

### Galvanic Vestibular Stimulation

Noisy GVS was applied with electrodes on the right and left mastoids by a linear isolator (DPS-560P/DPA-50, Physio-Tech, Tokyo, Japan) with digital storage for GVS waveforms, which are digital-to-analog converted at 20 Hz. Waveforms of noisy GVS and triggers for oVEMPs measurement were synthesized using MATLAB (MathWorks, Inc., Natick, MA, USA). We used band noise GVS ranging from 0.02 to 10 Hz. The waveform of noisy GVS was composed of band noise with a flat spectrum between 1 and 4000 Hz.

To reduce the artifact caused by GVS on the waveforms of oVEMPs, we inverted the waveform of band noise in the later half of the stimulus (from 5 to 8.5 s) from the one which was used in the early half of the protocol (from 0.5 to 4 s) (Figure 1). In each period, 15 triggers for generating BCV were applied at 5 Hz. By averaging all the responses of oVEMPs in the early and later periods, any GVS artifact contaminating oVEMP waveforms was reduced.

The root mean square (RMS) of the amplitude of GVS used was 0, 25, 100, 200, and 300 µA. The intensities were applied in a randomized order during oVEMP recording. During each stimulus, subjects were asked whether they feel any sensation or pain caused by noisy GVS.

### Data Analysis

We analyzed the first negative peak (N1) with a latency of around 10 ms and the second positive peak (P1). The latency was measured from the onset of the stimulus to the peak. The amplitude of N1 was measured from the baseline to the peak, and the amplitude of N1–P1 was measured between N1 and P1.

We judged that noisy GVS had facilitatory effects on oVEMP responses when it increased the amplitude of both N1 and N1–P1 simultaneously in at least two consecutive intensities of noisy GVS relative to the control (0 µA). The optimal noisy GVS stimulus was defined as that which led to the greatest increase in N1 amplitude.

Data are expressed as mean ± SEM. The ratio of each parameter during each intensity of noisy GVS to that without GVS (0 µA) was calculated [normalized ratio (NR)]. The NRs of oVEMP amplitude and latencies of the responses at each GVS amplitude were compared using a one-way repeated measures analysis of variance on ranks (RM ANOVA) followed by the Tukey *post hoc* test. The amplitudes and latencies of oVEMP responses measured during the control and optimal stimulation trials were compared using the Wilcoxon signed-rank test. A difference was considered significant at *p* < 0.05.

## Results

**Figure 2:**
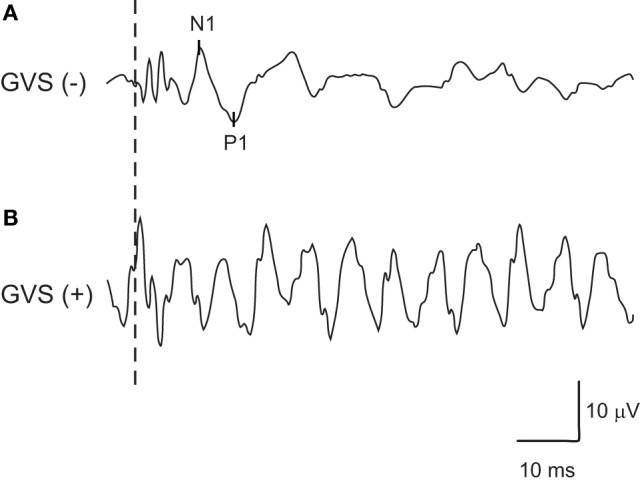
**An example of oVEMPs to bone-conducted vibration in which artifacts generated by noisy galvanic vestibular stimulation (GVS) could not be erased successfully by inverting the waveforms of noisy GVS in the later half of the stimulus from the one in the early half**. **(A)** oVEMP responses without GVS. **(B)** oVEMP responses with GVS.

By inverting the waveform of noisy GVS in the later half of stimulus to that in the early half, artifacts generated by GVS in the responses of oVEMPs to BCV were successfully erased in 34 (from 17 subjects) of the 40 ears (85%) in our 20 healthy volunteers (Figure 1). Six ears (from three subjects) in which artifacts generated by GVS could not be erased by this method were excluded from the analysis (Figure 2).

**Figure 3:**
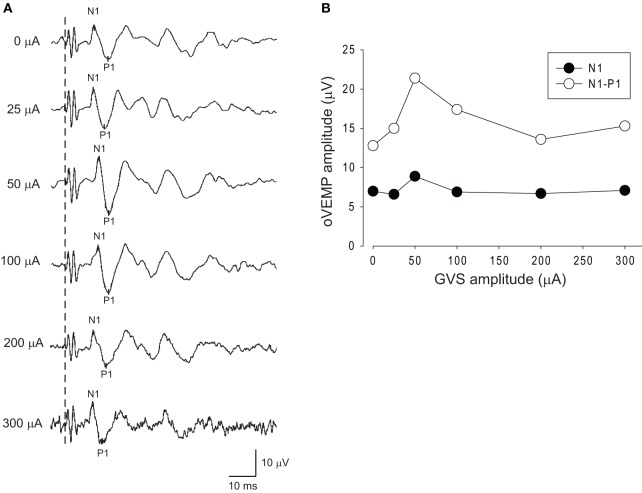
**Changes in oVEMPs to bone-conducted vibration (BCV) during application of noisy galvanic vestibular stimulation (GVS) in a representative subject**. **(A)** oVEMP responses to BCV during application of various intensities of GVS in a 58 year-old healthy subject. **(B)** Changes in the N1 and N1–P1 amplitudes of oVEMP responses during application of noisy GVS. In this subject, GVS at an intensity of 50 µA increased the amplitude of oVEMP responses, whereas further increases in intensity caused deterioration.

Figure 3 shows oVEMPs to BCV under various intensities of noisy GVS in a typical 58-year-old female subject. This subject showed an increase in the amplitude of both N1 and N1–P1 simultaneously under white noise GVS at an intensity of 50 µA, while a further increase in stimulus intensity resulted in a decrease in the amplitudes, suggesting the presence of an SR-like phenomenon (Figure 3).

**Figure 4:**
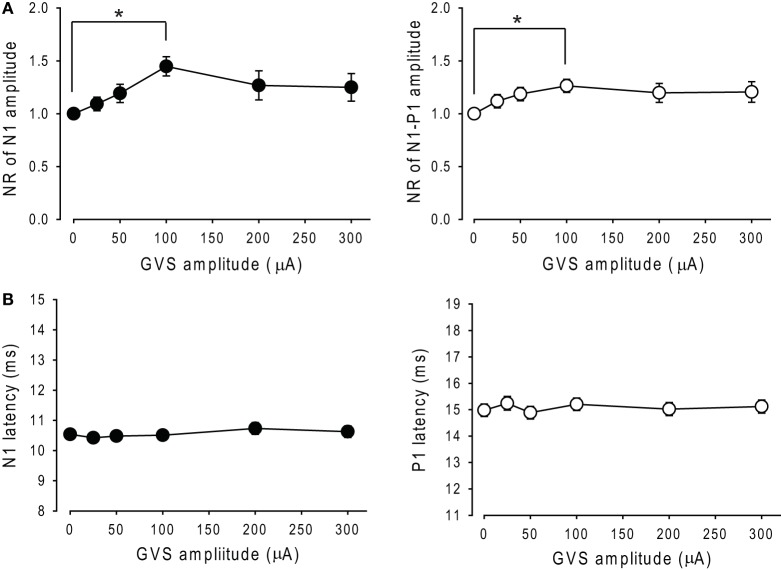
**Changes in the amplitude and latency of oVEMPs to bone-conducted vibration during application of noisy galvanic vestibular stimulation (GVS)**. **(A)** Changes in the normalized ratio of N1 amplitude (left panel) and N1–P1 amplitude (right panel) of oVEMP responses during application of noisy GVS in 34 ears. Mean ± 1 SEM is shown. *p < 0.05. **(B)** Changes in N1 latency (left panel) and P1 latency (right panel) of oVEMP responses during application of noisy GVS in 34 ears.

A comparison of NRs of N1 and N1–P1 amplitudes across each intensity of noisy GVS in 34 ears revealed that noisy GVS had significant effects on the amplitude of N1 as well as N1–P1 (RM ANOVA, *p* < 0.05; Figure 4A) and that there were significant differences between the control session (0 μA) and 100 µA GVS in both N1 and N1–P1 amplitudes (Tukey *post hoc* test, *p* < 0.05). On the other hand, noisy GVS had no significant effects on N1 or P1 latencies (RM ANOVA, *p* > 0.1; Figure 4B).

Among the 34 ears, noisy GVS had facilitatory effects in 27 ears (79%). The intensity of the optimal stimulus was 127 ± 14 µA, which was significantly smaller than the threshold of sensation (283 ± 21 µA; paired *t* test, *p* < 0.01). None of the subjects reported pain or unpleasant symptoms during or after the stimulus in this study.

**Figure 5:**
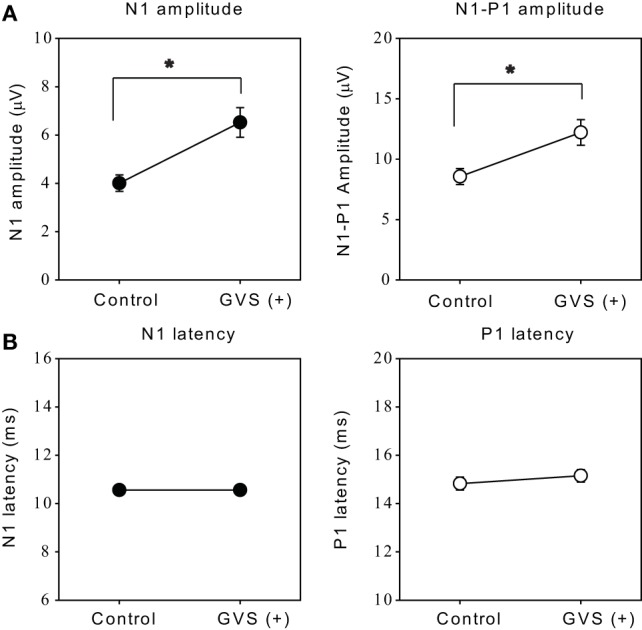
**Effects of the optimal intensity of noisy galvanic vestibular stimulation (GVS) on oVEMPs to bone-conducted vibration**. **(A)** Average (±1 SEM) of the N1 and N1–P1 amplitude without GVS (control) and with optimal intensity GVS [GVS (+)] across all ears in which noisy GVS had ameliorating effects (*n* = 27). *p < 0.05. **(B)** Average (±1 SEM) of the N1 and P1 latencies during control and GVS (+) across all ears in which noisy GVS had ameliorating effects (*n* = 27).

The optimal intensity of the stimulus increased N1 and N1–P1 amplitude by 75.9 ± 15% and 47.7 ± 9.1%, respectively, as compared with the 0 µA session (Wilcoxon signed-rank test, *p* < 0.05; Figure 5A). On the other hand, there were no significant effects on the latencies of N1 or P1 responses (*p* > 0.1; Figure 5B).

## Discussion

In the present study, we have examined the effects of low-intensity noisy GVS on oVEMPs to BCV in healthy subjects and have shown that an appropriate intensity of GVS can significantly increase the amplitude of oVEMP responses while it has no significant effects on their latencies.

Noisy GVS has been shown to improve static and dynamic postural stability, probably *via* SR (8, 16–20). We have previously shown that noisy GVS can improve postural stability in healthy individuals as well as in patients with bilateral vestibulopathy (8). Recently, Wuehr et al. have shown that noisy GVS is also able to improve dynamic postural stability during walking in healthy subjects (20) as well as in patients with bilateral vestibulopathy (19). However, it has not been tested whether noisy GVS can intensify vestibular function.

To examine the effect of noisy GVS on vestibular function, we employed oVEMPs to BCV. oVEMPs reflects the function of the utricle and its afferents mediated by a crossed-ocular pathway (14, 15). oVEMPs have several advantages over other vestibular function tests such as caloric tests or cervical vestibular-evoked vestibular myogenic potentials (21), one such advantage being that oVEMPs can be recorded easily and repeatedly without discomfort or fatigue. Since oVEMPs are excitatory myogenic potentials, they can detect subtle changes in amplification of vestibular responses as compared to cVEMPs in cases such as superior canal dehiscence syndrome (22) or Meniere’s disease (23).

However, we encountered a problem in recording oVEMPs under noisy GVS because GVS caused clear artifacts in the waveforms of the oVEMPs. We overcame this problem by inverting the waveform of noisy GVS in the second half of the stimulus. By averaging all the responses, we successfully reduced the GVS artifacts in the oVEMP waveforms in a majority of ears tested. The small number of ears in which artifacts could not be reduced might be due to insufficient lowering of skin resistance of the electrodes.

We applied various intensities of noisy GVS during the recording of oVEMPs to BCV and showed that an appropriate intensity of noisy GVS significantly increases the amplitude of the oVEMP responses in approximately 80% of healthy subjects. The hypothesized mechanism underlying this enhancement of oVEMP amplitude by noisy GVS is SR, in which an optimal amount of added noise results in enhancement of the information content whereas a further increase in the noise intensity degrades the content (9, 10). It has been shown that an appropriate intensity of noise can improve detection of weak subthreshold signals in visual (11, 24), auditory (12, 13), and tactile perception (25, 26). Since the vestibular system is fundamentally non-linear (27, 28), an appropriate intensity of noisy GVS might increase the activity of vestibular afferents by lowering the threshold of excitation through small changes in transmembrane potentials (3), leading to enhancement of oVEMP amplitudes. It is unknown why noisy GVS did not have facilitatory effects in approximately 20% of healthy subjects in this study. However, this result is consistent with our previous study that noisy GVS improved static postural stability in 76% of healthy subjects (8). It is possible that the subjects who did not show facilitatory effects of noisy GVS might already have sufficiently high vestibular function, so there might be little room for improving vestibular function.

In the present study, the optimal intensity of noisy GVS for oVEMPs to BCV was approximately 127 µA in RMS, which is smaller than the optimal intensity of the stimulus for improvement of postural stability in healthy subjects (228 µA in RMS) in our previous study (8). One possible explanation for this difference might be different sensitivity to noisy GVS between the vestibulo-ocular pathway and the vestibulo-spinal pathway. It has been shown that a higher stimulus intensity is necessary to evoke a vestibulo-spinal reflex in the leg than that in the neck (29). Another possibility is that there was a difference in the basal activities of the vestibular afferents between the oVEMPs and stabilometry recording sessions. In oVEMPs to BCV, noisy GVS modulates the activity of vestibular afferents, which are strongly activated by BCV, whereas in posturography, GVS modulates the activity of vestibular afferents, which are weakly activated by small head movements during two-legged stance tasks.

Our study has some limitations. First, we examined only oVEMPs to BCV to investigate the effect of noisy GVS on vestibular systems. It is possible that noisy GVS may have different effects on other vestibular function tests such as caloric tests or cervical VEMPs. To examine the effect of noisy GVS on vestibular afferents precisely, direct recording of the activity of vestibular afferents in animals is required (30). Second, artifacts caused by GVS could not be erased completely by reversing the noisy GVS stimulus during recording. Small artifacts of GVS which remained in oVEMP responses to BCV might have affected the precise measurement of the oVEMP responses. However, in oVEMP responses under noisy GVS up to 200 µA, the effect of artifacts was minimal in the present study. Third, we included healthy subjects only in this study. To confirm noisy GVS enhances the function of the vestibular afferents, it is favorable to include a group of patients with vestibular dysfunction. The experiment that examines the effect of noisy GVS on oVEMPs in patients with peripheral vestibular dysfunction will become the next step of our study.

In conclusion, we have shown that noisy GVS can increase the amplitude of oVEMPs to BCV in healthy subjects probably *via* SR. The results of the present study suggest that noisy GVS improves static and dynamic postural stability by enhancing the function of the vestibular afferents.

## Author Contributions

All the authors contributed the design of the work presented in this paper. SI, SK, and TK designed the experiment, gathered the data, performed the analysis, and wrote the manuscript. FT and CF designed the experiment, performed the analysis, supervised the writing, reviewed the manuscript, and edited the manuscript. YY and TY reviewed the manuscript and edited the manuscript. All the authors take full responsibility for the correctness of this paper and approved the final version.

## Conflict of Interest Statement

The authors declare that the research was conducted in the absence of any commercial or financial relationships that could be constructed as a potential conflict of interest.

## References

[1] Baloh RW Honrubia V Clinical Neurophysiology of the Vestibular System. 3rd ed New York: Oxford (2001). 378525

[2] Aw ST Todd MJ Aw GE Weber KP Halmagyi GM. Gentamicin vestibulotoxicity impairs human electrically evoked vestibulo-ocular reflex. Neurology (2008) 71(22):1776–82. 10.1212/01.wnl.0000335971.43443.d9 19029517

[3] Goldberg JM Smith CE Fernandez C. Relation between discharge regularity and responses to externally applied galvanic currents in vestibular nerve afferents of the squirrel monkey. J Neurophysiol (1984) 51(6):1236–56. 6737029 10.1152/jn.1984.51.6.1236

[4] Fitzpatrick RC Day BL. Probing the human vestibular system with galvanic stimulation. J Appl Physiol (2004) 96(6):2301–16. 10.1152/japplphysiol.00008.2004 15133017

[5] Soma R Nozaki D Kwak S Yamamoto Y. 1/f noise outperforms white noise in sensitizing baroreflex function in the human brain. Phys Rev Lett (2003) 91(7):078101. 10.1103/PhysRevLett.91.078101 12935054

[6] Pan W Soma R Kwak S Yamamoto Y. Improvement of motor functions by noisy vestibular stimulation in central neurodegenerative disorders. J Neurol (2008) 255(11):1657–61. 10.1007/s00415-008-0950-3 18677633

[7] Yamamoto Y Struzik ZR Soma R Ohashi K Kwak S. Noisy vestibular stimulation improves autonomic and motor responsiveness in central neurodegenerative disorders. Ann Neurol (2005) 58(2):175–81. 10.1002/ana.20574 16049932

[8] Iwasaki S Yamamoto Y Togo F Kinoshita M Yoshifuji Y Fujimoto C Noisy vestibular stimulation improves body balance in bilateral vestibulopathy. Neurology (2014) 82(11):969–75. 10.1212/WNL.0000000000000215 24532279

[9] Collins JJ Chow CC Imhoff TT. Stochastic resonance without tuning. Nature (1995) 376(6537):236–8. 10.1038/376236a0 7617033

[10] Moss F Ward LM Sannita WG. Stochastic resonance and sensory information processing: a tutorial and review of application. Clin Neurophysiol (2004) 115(2):267–81. 10.1016/j.clinph.2003.09.014 14744566

[11] Riani M Simonotto E Stochastic resonance in the perceptual interpretation of ambiguous figures: a neural network model. Phys Rev Lett (1994) 72(19):3120–3. 10.1103/PhysRevLett.72.3120 10056072

[12] Ries DT. The influence of noise type and level upon stochastic resonance in human audition. Hear Res (2007) 228(1–2):136–43. 10.1016/j.heares.2007.01.027 17350775

[13] Zeng FG Fu QJ Morse R. Human hearing enhanced by noise. Brain Res (2000) 869(1–2):251–5. 10.1016/S0006-8993(00)02475-6 10865084

[14] Iwasaki S McGarvie LA Halmagyi GM Burgess AM Kim J Colebatch JG Head taps evoke a crossed vestibulo-ocular reflex. Neurology (2007) 68(15):1227–9. 10.1212/01.wnl.0000259064.80564.21 17420408

[15] Iwasaki S Smulders YE Burgess AM McGarvie LA Macdougall HG Halmagyi GM Ocular vestibular evoked myogenic potentials to bone conducted vibration of the midline forehead at Fz in healthy subjects. Clin Neurophysiol (2008) 119(9):2135–47. 10.1016/j.clinph.2008.05.028 18639490

[16] Mulavara AP Fiedler MJ Kofman IS Wood SJ Serrador JM Peters B Improving balance function using vestibular stochastic resonance: optimizing stimulus characteristics. Exp Brain Res (2011) 210(2):303–12. 10.1007/s00221-011-2633-z 21442221

[17] Pal S Rosengren SM Colebatch JG. Stochastic galvanic vestibular stimulation produces a small reduction in sway in Parkinson’s disease. J Vestib Res (2009) 19(3–4):137–42. 10.3233/VES-2009-0360 20448339

[18] Pavlik AE Inglis JT Lauk M Oddsson L Collins JJ. The effects of stochastic galvanic vestibular stimulation on human postural sway. Exp Brain Res (1999) 124(3):273–80. 10.1007/s002210050623 9989432

[19] Wuehr M Nusser E Decker J Krafczyk S Straube A Brandt T Noisy vestibular stimulation improves dynamic walking stability in bilateral vestibulopathy. Neurology (2016) 86(23):2196–202. 10.1212/WNL.0000000000002748 27164706

[20] Wuehr M Nusser E Krafczyk S Straube A Brandt T Jahn K Noise-enhanced vestibular input improves dynamic walking stability in healthy subjects. Brain Stimul (2016) 9(1):109–16. 10.1016/j.brs.2015.08.017 26422129

[21] Welgampola MS Colebatch JG Characteristics and clinical applications of vestibular-evoked myogenic potentials. Neurology (2005) 64(10):1682–8. 10.1212/01.WNL.0000161876.20552.AA 15911791

[22] Manzari L Burgess AM McGarvie LA Curthoys IS. Ocular and cervical vestibular evoked myogenic potentials to 500 Hz fz bone-conducted vibration in superior semicircular canal dehiscence. Ear Hear (2012) 33(4):508–20. 10.1097/AUD.0b013e3182498c09 22441357

[23] Manzari L Tedesco AR Burgess AM Curthoys IS Ocular and cervical vestibular-evoked myogenic potentials to bone conducted vibration in Meniere’s disease during quiescence vs during acute attacks. Clin Neurophysiol (2010) 121(7):1092–101. 10.1016/j.clinph.2010.02.003 20202901

[24] Kitajo K Nozaki D Ward LM Yamamoto Y. Behavioral stochastic resonance within the human brain. Phys Rev Lett (2003) 90(21):218103. 10.1103/PhysRevLett.90.218103 12786595

[25] Collins JJ Imhoff TT Grigg P. Noise-enhanced information transmission in rat SA1 cutaneous mechanoreceptors via aperiodic stochastic resonance. J Neurophysiol (1996) 76(1):642–5. 8836253 10.1152/jn.1996.76.1.642

[26] Ivey C Apkarian AV Chialvo DR. Noise-induced tuning curve changes in mechanoreceptors. J Neurophysiol (1998) 79(4):1879–90. 9535955 10.1152/jn.1998.79.4.1879

[27] Minor LB Lasker DM Backous DD Hullar TE. Horizontal vestibuloocular reflex evoked by high-acceleration rotations in the squirrel monkey. I. Normal responses. J Neurophysiol (1999) 82(3):1254–70. 10482745 10.1152/jn.1999.82.3.1254

[28] Sadeghi SG Chacron MJ Taylor MC Cullen KE. Neural variability, detection thresholds, and information transmission in the vestibular system. J Neurosci (2007) 27(4):771–81. 10.1523/JNEUROSCI.4690-06.2007 17251416 PMC5053814

[29] Welgampola MS Colebatch JG. Selective effects of ageing on vestibular-dependent lower limb responses following galvanic stimulation. Clin Neurophysiol (2002) 113(4):528–34. 10.1016/S1388-2457(02)00020-2 11955997

[30] Minor LB Goldberg JM. Vestibular-nerve inputs to the vestibulo-ocular reflex: a functional-ablation study in the squirrel monkey. J Neurosci (1991) 11(6):1636–48. 2045879 10.1523/JNEUROSCI.11-06-01636.1991 PMC6575423

